# Editorial: Biological and digital markers in sleep, circadian rhythm and epilepsy using artificial intelligence

**DOI:** 10.3389/fphys.2025.1616497

**Published:** 2025-05-12

**Authors:** Haoqi Sun, Felicia Jefferson, Ankit Parekh, Margarita Oks, Cathy Goldstein

**Affiliations:** ^1^ Department of Neurology, Beth Israel Deaconess Medical Center, Boston, MA, United States; ^2^ Department of Biochemistry and Molecular Biology, University of Nevada, Reno, NV, United States; ^3^ Division of Pulmonary, Critical Care and Sleep Medicine, Department of Medicine, Icahn School of Medicine at Mount Sinai, New York, NY, United States; ^4^ Northwell Health, New York, NY, United States; ^5^ Department of Neurology, University of Michigan, Ann Arbor, MI, United States

**Keywords:** sleep, artificial intelligence, digital markers, sleep apnea, brain aging

Sleep is critical to many organ systems, including the brain (Gottesman et al., 2024), cardiovascular system (Malhotra and Loscalzo, 2009), metabolism (Feeney et al., 2025), and immune system (Irwin, 2019). Therefore, recording the sleep state provides rich physiological data, allowing a window into health and disease (Sun et al., 2024). Polysomnogram is the cornerstone of sleep diagnostics; therefore, the sleep field has amassed many multidimensional, time series electrophysiological data (Zhang et al., 2018; Zhang et al., 2024). Additionally, given the ubiquity of multi-sensor consumer sleep tracking devices, longitudinal data comprised of various signal modalities is passively collected during sleep in the home environment at scale. Therefore, artificial intelligence (AI) is well-suited to discover biological and digital markers that track and predict health conditions (Bandyopadhyay and Goldstein, 2023; Bandyopadhyay et al., 2024; Sun et al., 2025).

Here, we discuss four studies on this Research Topic. Each addresses a key question: making sleep staging more generalizable across different settings from Van Der Aar et al., improving home diagnosis of sleep apnea with autonomic signals from Ross et al., extending sleep staging to children with epilepsy from Proost et al., and using sleep electroencephalography (EEG) to measure brain aging in people with epilepsy from Hadar et al.

Sleep staging AI models trained on a limited number of data sets may demonstrate reduced effectiveness when deployed across diverse demographics and recording settings. Pre-trained models, especially foundation models (Thapa et al., 2024; Moor et al., 2023), serve as feature extractors but require specialized heads to adapt to different tasks. Van Der Aar et al. analyzed strategies to mitigate these mismatches using deep transfer learning. They found that fine-tuning often has a higher accuracy with fewer target data, improving Cohen’s kappa by 0.15 in patients with REM sleep behavior disorder (RBD). However, targeted training was necessary using different signal channels, leading to an average Cohen’s kappa improvement of 0.17. The authors concluded that pre-training extensive datasets with specific adaptations enables robust sleep staging for diverse populations and recording scenarios. Their results align with other transfer learning studies in sleep research (Ganglberger et al., 2024; Radha et al., 2021; Olsen et al., 2024). While fine-tuning necessitates labeled data from the new domain, this study outlines an essential framework for heterogeneous data sources.

Home sleep apnea testing (HSAT) has replaced a large portion of laboratory testing to diagnose obstructive sleep apnea (OSA), given its convenience. However, HSATs do not stage sleep as defined by EEG, and they often use recording time as the denominator to calculate the apnea-hypopnea index (AHI). This practice leads to underestimating the AHI and falsely negative HSAT results. Ross et al. developed an AI model to infer sleep stages and arousal from heart rate and breathing using 245 participants (148 with HSATs). Incorporation of objective sleep estimates into the AHI improved obstructive sleep apnea classification from 70% to 80% and reduced AHI underestimation from 19% to 7%, without losing specificity. The results support reliable and portable diagnostics, potentially identifying more mild-to-moderate cases of obstructive sleep apnea outside of the sleep laboratory.

Another challenge in accurate sleep analysis is the confounding effect of diseases, such as epilepsy, according to Proost et al. Sleep and epilepsy form a vicious cycle (Maganti and Jones, 2021; Halász and Szűcs, 2020): epileptic discharges disrupt sleep, resulting in fragmentation, reduced slow wave activity (SWA), and reduced spindle density. Conversely, apnea and sleep instability can trigger seizures, leading to abnormal K-complexes or severe electrical status epilepticus of sleep. Proost et al. studied 176 children aged 4–18  years with drug-resistant epilepsy (DRE) and well-controlled epilepsy (WCE). They addressed two key gaps: the absence of automated sleep analysis in pediatric epilepsy and the feasibility of minimal electrode use. However, following the findings from Van Der Aar et al., the model should be tested for accuracy in at-home settings using wearable EEGs for long-term monitoring. This would enable tracking sleep quality over multiple nights, correlate with seizure patterns, and enhance sleep management in epilepsy care.

Beyond sleep staging, sleep can reflect brain aging. Hadar et al. employed the sleep EEG-based brain age index (BAI) (Sun et al., 2019), an AI-driven metric integrating age-dependent changes in sleep, quantifying how much the sleep EEG deviates from the chronological age. A positive BAI indicates an “older” brain. The hypothesis is that neurological stress from epilepsy is associated with accelerated brain aging. They studied 138 epilepsy patients and age-matched controls, finding that those with epilepsy had a BAI of about +5 years, while healthy participants had a near-zero BAI. The results support the hypothesis, especially in patients with severe epilepsy: generalized seizures had a higher BAI (5.5 years older) than focal seizures (3.3 years older). While this study cannot prove causality, it suggests AI analysis of sleep EEG may serve as brain health markers.

However, these papers do not comprehensively address the Research Topic, particularly the molecular markers. While various omics studies of health outcomes exist, the (multi-)omic bases of sleep are still unexplored. Since sleep and circadian rhythms are vital to many biological functions, we anticipate meaningful omic biomarkers linked to sleep patterns that indicate functional and health outcomes. For example, BACE1 was found to promote the cleavage of the GABA_A_ receptor and contribute to neural hyperexcitability in Alzheimer’s disease (Bi et al., 2025), which likely alters sleep EEG microstructures. The omic biomarkers could serve as potential intervention targets to enhance sleep physiology and outcomes. Another limitation is the inability of the discussed methods to account for cause-and-effect relationships between sleep disturbances and diseases. Although the big datasets in the sleep and circadian fields are critical for AI development (Zhang et al., 2018; Zhang et al., 2024), many are cross-sectional and do not support causal inference. We encourage longitudinal studies and novel applications of causal discovery to understand the causal nature, thus providing insights into mechanisms and decision-making. Finally, multimodal data integration should be used. Sleep and circadian systems demonstrate multi-organ interactions. For example, sleep apnea has consequences across multiple organ systems (Azarbarzin et al., 2024), including oxygen levels (Azarbarzin et al., 2019), heart rate (Azarbarzin et al., 2021), and cortical arousal (Eckert and Younes, 2014). Therefore, multimodal wearable technologies comprised of sensors that monitor respiratory function, cardiac autonomic activity, motion and body position, and temperature will enable real-time, longitudinal tracking of sleep and circadian parameters simultaneously with other physiological processes associated with disease states. In summary, utilizing such AI-based sleep biomarkers will allow the integration of sleep data into comprehensive health assessments.
